# Improved Dry Eye Symptoms and Signs of Patients With Meibomian Gland Dysfunction by a Dietary Supplement

**DOI:** 10.3389/fmed.2021.769132

**Published:** 2021-11-12

**Authors:** Kangcheng Liu, Yau Kei Chan, Xia Peng, Ruolan Yuan, Min Liao, Jingwen Liang, Xiangbo Tang, Yi Xu, Yingjun Cai, Qiangxiang Li, Hua Wang

**Affiliations:** ^1^Eye Center of Xiangya Hospital, Hunan Key Laboratory of Ophthalmology, Central South University, Changsha, China; ^2^Department of Ophthalmology, Li Ka Shing Faculty of Medicine, The University of Hong Kong, Hong Kong, China; ^3^Ningxia Geriatric Disease Clinical Research Center, People's Hospital of Ningxia Hui Autonomous Region, Yinchuan, China

**Keywords:** dietary supplements, meibomian gland dysfunction (MGD), dry eye, Retaron^®^, symptoms and signs

## Abstract

**Purpose:** To explore the therapeutic effect of a dietary supplement on dry eye with meibomian gland dysfunction (MGD).

**Methods:** Sixty patients with MGD-related dry eye were included in this prospective and randomized, placebo-controlled study. All the subjects were treated with eye hot compress, artificial tears, and antibiotic ointment. After that, the patients received dietary supplementary or placebo daily for 12 weeks. The dry eye signs, function of MG, and visual quality of the patients were assessed at 4, 8, and 12 weeks after the treatment.

**Results:** Twelve weeks after the treatment, patients who received dietary supplement had a significantly better improvement of dry eye symptoms, in terms of ocular surface diseases index and tear breaking-up time (TBUT), than those who received placebo (*P* < 0.05). The functions of MG, in terms of meibum quality and MG exclusion and MG obstruction scores, were significantly improved in both dietary supplement and placebo groups (*P* < 0.05). Patients who received dietary supplement had a significantly better improvement in the MG structure, in terms of acinar diameter and acinar density, than those who received placebo (*P* < 0.05). The number of inflammatory cells near MG was significantly lower in the dietary supplement group when compared with the placebo group (*P* < 0.05). The objective visual quality was significantly improved in the dietary supplement group, but not in the placebo group (*P* < 0.05).

**Conclusion:** The dietary supplement can effectively improve the symptoms and signs of MGD-related dry eye, reduce the inflammatory reaction of MG, restore the gland structure, and indirectly improve the visual quality.

## Introduction

Meibomian gland dysfunction (MGD) is a chronic, diffuse meibomian gland lesion. The main features of MGD are the obstruction of the terminal duct of the meibomian glands and the abnormal quality or quantity of the meibum secretion (1). This disease causes tear film abnormalities with inflammatory reactions, resulting in eye irritation. The cornea gets damage and visual function is affected in severe MGD (1). When MGD occurs, the change of meibum, the main component of the lipid layer of the tear film, affects the steady state of the tear film and results in evaporative dry eye. Since the publication of the Tear Film and Ocular Surface Society Dry Eye Workshop (TFOS DEWS) report, numerous studies have conducted to investigate the correlation between MGD and dry eye (2). MGD is believed to be the most common cause of dry eye (3, 4). Epidemiological surveys of MGD patients from different ages, genders, and races showed that the prevalence of MGD over 40 years is from 38 to 68% (5–7). Other epidemiological surveys based on different ethnic groups have found that around 53.6–89.7% of patients in the dry eye population are with MGD (8, 9). Therefore, the functional improvement of MG becomes the major strategy to treat MGD-related dry eye patients (10).

The current treatment of MGD-related dry eye includes physical and oral drug therapies, such as eye drops, hot eye compresses, and MG massage (11). However, these treatments also have own limitations. Hot eye compresses need to maintain a constant temperature to liquefy the secretions that block MG, which is troublesome in real practice (12, 13). Similarly, although MG massage has a better theoretical therapeutic effect, it is easy to cause damage to the MG or cornea, and more severely secondary infection. Moreover, patient compliance is poor (14). At present, systemic oral bioactives, such as various vitamins and antibiotics, are needed for patients with severe MGD or MGD with seborrheic dermatitis, rosacea, and other skin lesions (15). As patients require a long-term use of antibiotics, they may suffer from the emergence of drug-resistant bacteria, bacterial imbalance and double infections, and other serious side effects (15). Meanwhile, among the commercially available drugs that treat dry eye diseases, most of them have poor acceptance from the stakeholders. Therefore, it is of great importance to find a bioactive that can safely and effectively treat MGD-related dry eye.

Dietary supplements have been widely used as an adjunct therapy for patients with various diseases (16). Many clinical studies have shown that dietary supplements can safely and effectively be used as adjuvant therapy (17). The American epidemiological survey shows that as many as 53% of the U.S. population have routine intake of dietary supplements, showing a very high population acceptance to the dietary supplements (18). The identification of dietary supplements that can treat MGD-related dry eye safely and effectively will have important clinical value and significance. Retaron^®^ is a new dietary supplement formulation on the market that has recently obtained the national safety standard identification in China. Its main ingredients include omega-3 free fatty acids, lutein, aronia extract, vitamin C, and vitamin E. It has potent antioxidant and anti-inflammatory effects, which play an important role in promoting retinal development and improving visual function (19). In this study, we investigated the effect of the dietary supplement as an adjunct treatment of MGD-related dry eye. Our hypothesis was that the use of dietary supplement can improve dry eye symptoms and signs, reduce the inflammatory reaction of MG, and restore the gland structure in patients with MGD.

## Materials and Methods

### Study Population

Between September 2018 and May 2019, 60 patients with binocular MGD-related dry eye were recruited from the ocular surface specialist outpatient clinic at Xiangya Hospital Ophthalmology Center. The diagnostic criteria of MGD and dry eye are based on Chinese Expert Consensus on the Diagnosis and Treatment of MGD (2017) (20) and TFOS DEWS II (2017) (21).

The inclusion criteria include (1) the age of patients in between 18 and 80 years and (2) the best-corrected visual acuity in both eyes that is higher than 0.1. The exclusion criteria include (1) systemic diseases such as systemic lupus erythematosus, rheumatism, and diabetes; (2) history of eye surgery and trauma; (3) other eye diseases; (4) systemic and ocular medications; (5) pregnant or lactating women; (6) patients who are allergic to the ingredients of the dietary supplement formulation or the drugs involved in this study; and (7) people who cannot take care of themselves and cooperate with treatment and follow-up.

### Informed Consent

All patients have agreed and signed the consent form to be included in the study. This study followed the Declaration of Helsinki, conformed to the principles of medical ethics, and was approved by the Ethics Committee of Xiangya Hospital of Central South University (Document number: 201912528).

### Study Design

The MGD-related dry eye patients included in this study were randomly divided into two groups, the experimental dietary supplement group (60 eyes of 30 cases) and placebo control group (60 eyes of 30 cases). Both the groups were respectively treated with oral dietary supplementary formulation Retaron^®^ (Table 1) and placebo capsule daily for 12 weeks. The eyelid margins of all patients were applied with tobramycin and dexamethasone ophthalmic ointment (once per night) for the first 2 weeks, followed by levofloxacin ophthalmic gel (once per night) for the next 10 weeks. Eye hot compress (once per night) and eye drops of 0.1% sodium hyaluronate (four times a day) were applied in adjunct with the dietary supplement for 12 weeks.

**Table 1:** Formula of dietary supplement Retaron^®^ in the study.

**Formula**	**Retaron^®^**
β-Carotene	–
Lutein	10 mg
Zeaxanthin	2 mg
Vitamin C	100 mg
Vitamin E	20 mg
Zinc	10 mg
Copper	–
Selenium	25 μg
Taurine	50 mg
Aronia extract	50 mg
Omega-3 free fatty acids, 250 mg DHA/30 mg EPA	500 mg

### Clinical Examinations

All subjects were followed up for 12 weeks with a 4-week interval. To avoid systematic errors, all the inspections and operations described below were completed by the same experienced ophthalmologist or technician. All subjects underwent routine examinations, such as vision, optometry, intraocular pressure, and fundus, at each follow-up.

Dry eye symptoms and signs were evaluated by Ocular Surface Disease Index (OSDI) Questionnaire Scale, (20) tear breaking-up time (TBUT), and corneal fluorescein (FL) staining. TBUT is an index used to evaluate the stability of the tear film. For each patient at each time point, TBUT was obtained by averaging the values from three measurements. TBUT value > 10 s is regarded as normal. Corneal FL staining helped us to examine the severity of corneal epithelial damage and was quantitatively evaluated using the 4-quadrant 12-point method as previously described (22).

The MG obstruction (23) of each eyelid was evaluated using a slit lamp microscope and anterior segment camera system with the following self-defined scoring system (24): 0 point, unobstructed opening; 1 point, MG with <1/3 obstruction; 2 points, between 1/3 and 2/3 obstruction; and 3 points, over 2/3 obstruction.

All the five MGs in the upper eyelid of each subject were squeezed, and the function of MG was assessed by the following MG discharge capacity score (20): 0 point, all five glands have discharge; 1 point: three or four glands have discharge; 2 points, only one or two glands have discharge; and 3 points, no gland with discharge. The meibum was evaluated according to the viscosity and turbidity of meibum using the meibum quality score: (20) 0 point, clear, transparent liquid; 1 point, turbid liquid; 2 points, turbid granular discharge; and 3 points, thick discharge like toothpaste.

The acinar morphology and inflammation of MG were examined using *in vivo* confocal microscopy (IVCM) (Heidelberg, Germany) and evaluated by (1) the diameter of MG opening, (2) acinar diameter, (3) acinar density, and (4) local inflammatory cell count (25, 26). For each parameter, ten photographs were randomly evaluated, and the average value of the ten measurements of the four previously mentioned parameters was recorded.

The visual quality of the patients was objectively assessed using the Optical Quality Analysis System (Visiometrics, Spain). Several visual quality assessment parameters were assessed, including modulation transfer function cutoff frequency (MTF_cutoff_), Strehl ratio (SR), and objective scatter index (OSI). MTF_cutoff_ reflects the resolution of the optical system, and its value is directly proportional to the visual quality. The value below 30 c/deg is considered as abnormal visual quality. SR is the ratio of the light intensity at the Gaussian image point of the actual refractive medium to the light intensity at the Gaussian phase point of the normal refractive medium. The higher the SR value, the closer it is to an aberration-free optical system. Below 0.15, the visual quality is considered abnormal. OSI reflects the degree of turbidity of the refractive medium. Values higher than 2.0 are regarded as abnormal, and higher values represent more turbid refractive media.

### Statistical Analysis

Statistical analysis was performed using SPASS 19.00 (IBM Corp, Armonk, NY, United States). The Kolmogorov–Smirnov test was used to test the normality of the data. Normally distributed data were expressed as mean ± SD, and non-normally distributed data were expressed as median and interquartile range. If the data conformed to be normally distributed, the comparisons between groups at each time point were tested by repeated-measures ANOVA (multiple time points) and independent-sample *t*-test (two time points). If the data did not meet the “spherical symmetry” assumption, the Greenhouse–Geisser estimate was used for correction. The LSD *t*-test (multiple time points) and paired *t*-test (two time points) were used for group comparison at each time point. If the data did not conform to be normally distributed, the Mann–Whitney *U*-test and the Kruskal–Wallis test were used for pairwise comparisons between groups at each time point. The test level was α = 0.05, and *P* < 0.05 was considered statistically significant.

## Results

### Clinical Outcomes

A total of 60 subjects (120 eyes) were included in this study, namely, 30 subjects (60 eyes) in the dietary supplement group and 30 subjects (60 eyes) in the control group. Before the treatment, there were no significant differences in age, best-corrected visual acuity, or intraocular pressure between the two groups (*P* > 0.05 for all indices) (Table 2).

**Table 2:** Comparison of the demography of the two groups of patients.

	**Range**	**Dietary supplement group**	**Control group**	***P***
Sex (Male/female)	/	10/14	13/17	N/A
Age (years)	18–80	44.23 ± 14.03	46.83 ± 12.03	0.444
BCVA^U^	0.1–1.5	1.2 (1.0, 1.2)	1.2 (1.0, 1.2)	0.954
IOP (mmHg)	10–21	17.10 ± 2.07	16.03 ± 2.31	0.065

U *Data are tested by Mann–Whitney U-test*.

*BCVA, best-corrected visual acuity; IOP, intraocular pressure*.

### Dry Eye Symptoms and Signs

**Figure 1:**
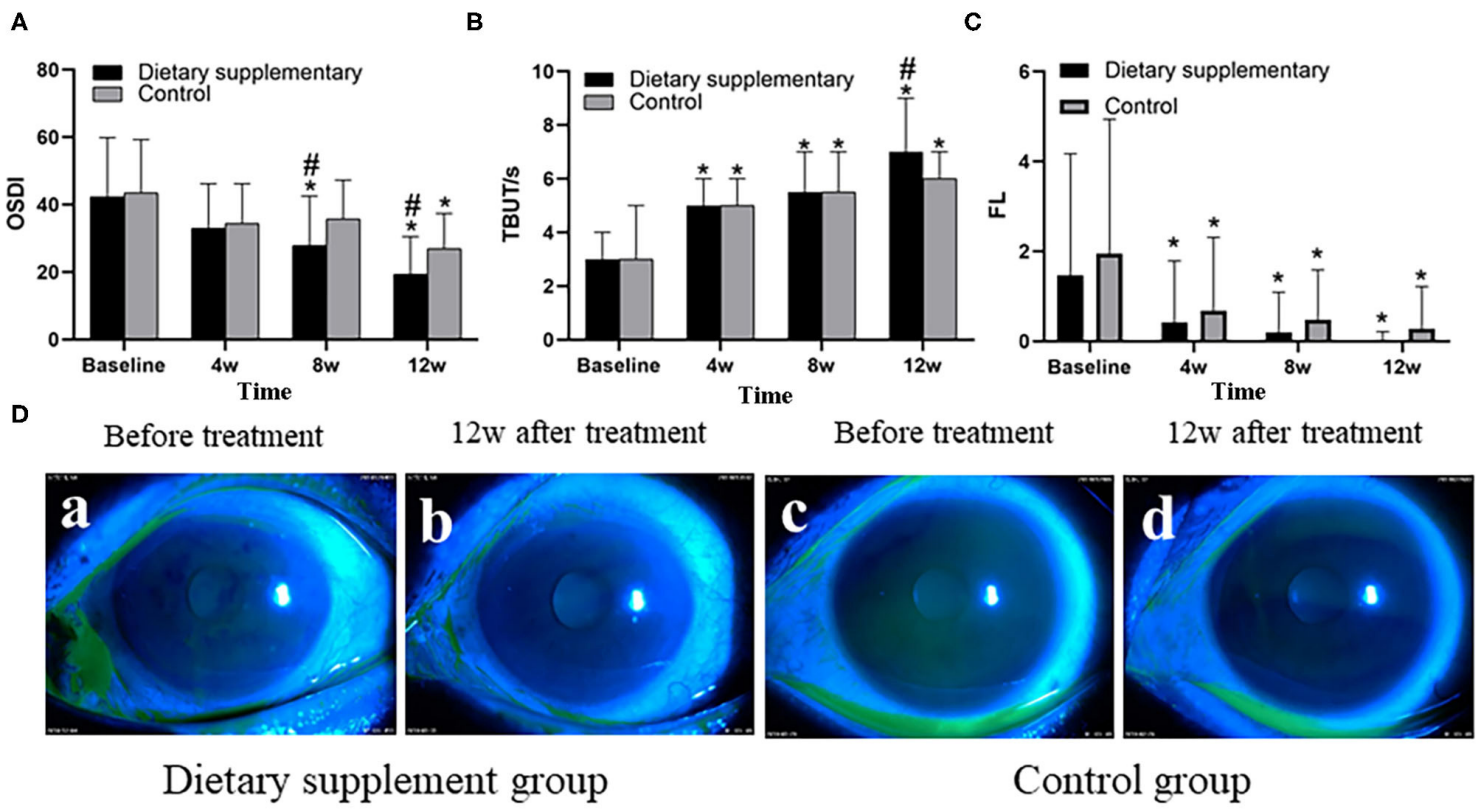
The **(A)** OSDI, **(B)** TBUT, and **(C)** FL of the two groups. **(D)** Anterior segment images of corneal FL staining between the two groups before and after the treatment. ^*^P < 0.05 vs. before the treatment; ^#^*P* < 0.05 vs. control group.

Before the treatment, there was no significant difference between the two groups in terms of the symptoms and signs of dry eye as quantified by OSDI and FL scores and TBUT (*P* > 0.05 for all indices). Twelve weeks after the treatment, punctate staining of corneal epithelium disappeared in both groups (Figure 1D), and both the OSDI and FL scores of the two groups of subjects were significantly lower than the baseline values (*P* < 0.001) (Figures 1A,C). However, TBUT score of the two groups was significantly higher than the baseline values (*P* < 0.001) (Figure 1B). Moreover, the dietary supplement group showed better outcomes, with lower OSDI and FL scores, and a higher TBUT, when compared with the control group (*P* < 0.05 for all indices) (Figure 1).

### The Function of MG and MG Obstruction

**Figure 2:**
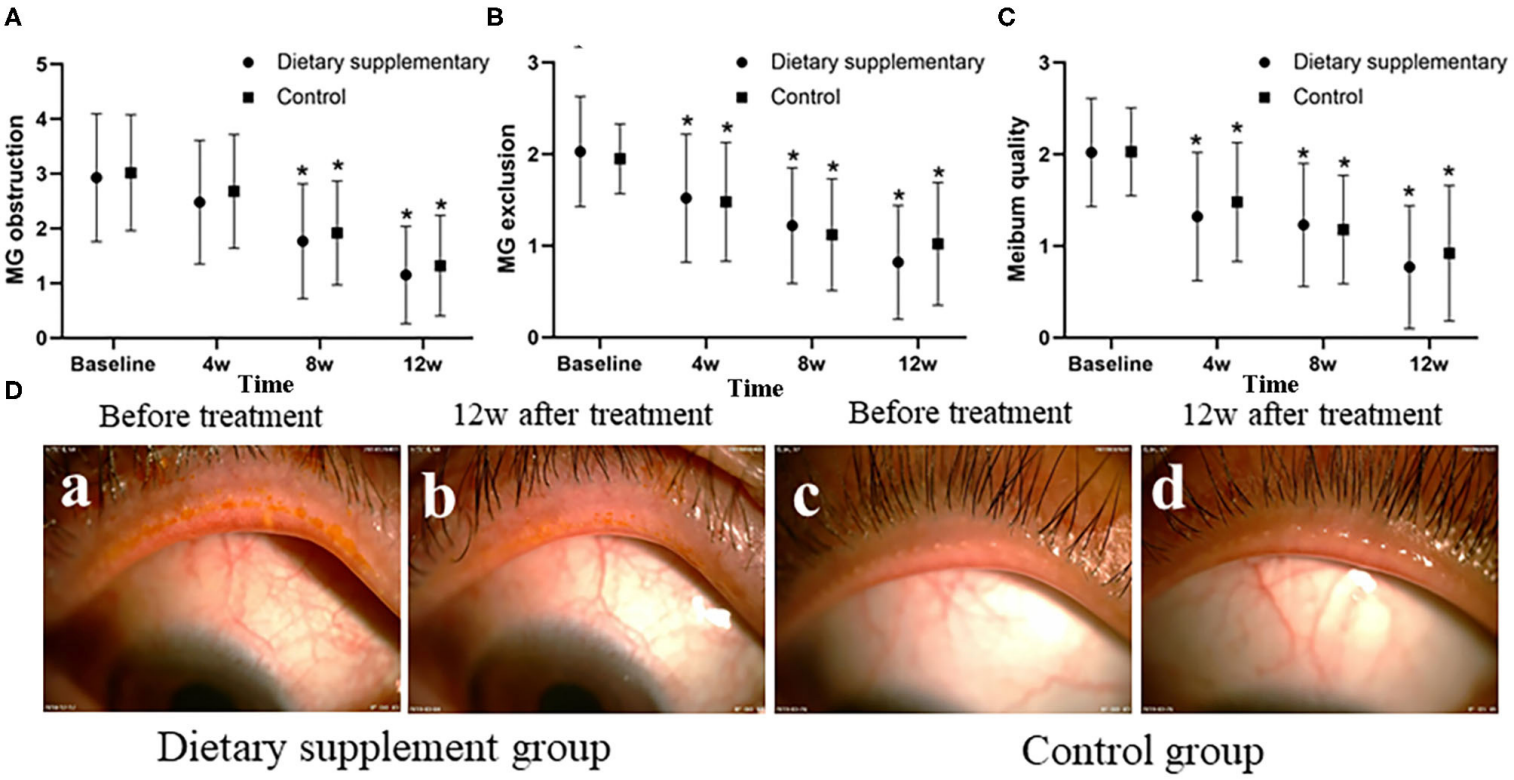
The function of MG and MG obstruction between two groups. **(A)** The MG obstruction score, **(B)** the MG exclusion, and **(C)** the meibum quality score. **(D)** Anterior segment images of palpebral margin between the two groups before and after the treatment. ^*^P < 0.05 vs. before the treatment.

Before the treatment, the upper eyelid margin congestion and MG obstruction were significant in both groups, and the meibum was viscous and turbid and difficult to be excreted when the MG was compressed (Figure 2D-a,c). There was no difference between the two groups in MG obstruction, MG discharge function, and meibum quality (*P* > 0.05 for all indices). After 12 weeks of treatment, the upper eyelid margin congestion was reduced in both groups, and MG obstruction was significantly improved. The meibum was also clear and easy to be excreted when the MG was compressed (Figure 2D-b,d). The MG exclusion score, meibum quality score, and the obstruction score of the two groups were significantly improved after the treatment at 8 and 12 weeks (*P* ≤ 0.001 for all time points). However, there is no significant difference between the two groups at the same time point (*P* > 0.05 for all indices) (Figures 2A–C).

### Morphological Structure of MG and Local Inflammatory Response

**Figure 3:**
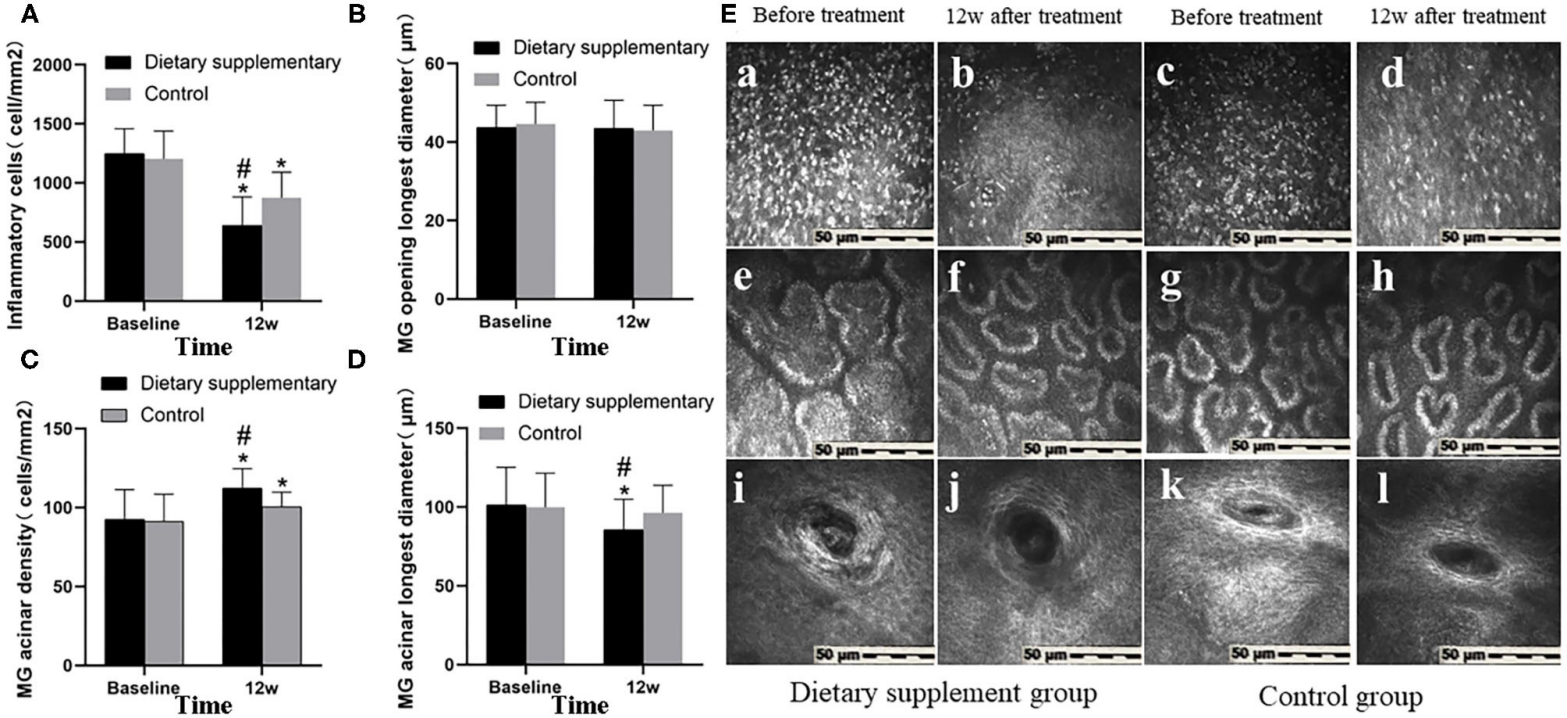
The IVCM between two groups. **(A)** Inflammatory cell number. **(B)** The longest diameter of MG opening. **(C)** MG acinar density. **(D)** MG acinar longest diameter. **(E)** IVCM images of MG before and after treatment in both groups. (a–d) Inflammatory cells. (e–h) Acinar morphology and density. (i–l) The shape of MG opening. ^*^P < 0.05 vs. before treatment; ^#^*P* < 0.05 vs. control group.

Before the treatment, there was no significant difference between the two groups in inflammatory cell number (Figures 3A,E-a,c), MG opening diameter (Figures 3B,E-i,k), acinar density (Figures 3C,E-e,g), and acinar diameter (Figures 3D,E-e,g) (*P* > 0.05 for all indices). Twelve weeks after the treatment, the number of inflammatory cells (Figures 3A,E-b), acinar density (Figures 3C,E-f), and acinar diameter (Figures 3D,E-f) in the dietary supplement group were significantly improved (*P* ≤ 0.001 for all indices) and were significantly better than in the control group (*P* ≤ 0.001 for all indices) (Figures 3A,C–E-d,h). The diameter of MG in both groups was not significantly improved when compared with the baseline level (*P* > 0.05 for two groups) (Figures 3B,E-j,l).

### Objective Visual Quality

**Figure 4:**
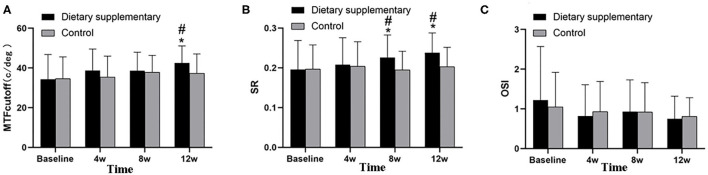
Objective visual quality between two groups. **(A)** MTF_cutoff_, **(B)** SR, and **(C)** OSI. ^*^P < 0.05 vs. before treatment; ^#^*P* < 0.05 vs. control group.

Before the treatment, there was no significant difference in the three visual quality parameters, namely, *P*_*MTFcutof*_, *P*_*SR*_, and *P*_*OSI*_, between the two groups (*P* > 0.05 for all indices). Twelve weeks after the treatment, the MTF_cutoff_ and SR of the dietary supplement group were significantly improved (*P* < 0.001 for MTF_cutoff_ and SR). In contrast, the improvement of the control group, when compared with the baseline, was not significant (*P* > 0.05 for MTF_cutoff_ and SR). Moreover, the difference between the two groups was significant (*P*_*MTFcutoff*_ = 0.003, *P*_*SR*_ < 0.001) (Figures 4A,B). However, there was no significant difference in OSI between two groups (*P*_*OSI*_ > 0.05 for two groups) (Figure 4C).

### Adverse Reactions

During the follow-up, no obvious local or systemic adverse reactions were observed in all subjects.

## Discussion

The MG is a special exocrine gland. The meibum secreted by the acinar epithelium forms a lipid layer on the tear film surface to make the tear film stable. When the MG is blocked or atrophic, this causes pathological changes in both the quality and quantity of the meibomian ester and its excretion, and finally leads to MGD. At the same time, MGD is often accompanied by local inflammatory reaction, which will eventually lead to lipid deficiency-type dry eye. Current studies have confirmed that MGD is one of the most important causes of dry eye (4).

Dietary supplements have been widely used for long time in all age groups worldwide, especially in developed countries, where dietary supplement use is as high as 11.7–66% (27). It plays an important role in balancing the nutrition of the normal population and the auxiliary treatment of the sick population, and it has been proved safe and effective. In our study, dietary supplement was used as an adjunct therapy to treat and to explore the safety and efficacy on MGD-related dry eye. Our results indicated that the adjunct therapy using dietary supplement can improve the ocular symptoms and signs, MG structure, local inflammatory response, and objective visual quality of MGD-related dry eye patients, but no significant advantage in the improvement of the properties and excretion capacity of meibum.

Meibomian gland produces and secretes meibomian ester in a constant manner, which makes it a high oxygen consumption tissue. Long-term and excessive oxidative reaction is an important reason for the dysfunction of MG. Oxidative stress is often accompanied by local inflammation of the MG. The clinical manifestations are mucous and muddy meibum, abnormal increase or decrease in secretion, gland obstruction and pathological dilatation, ester embolization obstruction at the opening, congestion and edema at the edge of eyelid, and neovascularization, which may lead to lipid deficiency-type dry eye (28). Our study showed that the symptoms and signs of the dry eye, MG structure, local inflammatory response, and objective visual quality were all significantly improved after the treatment. The possible mechanism is that the active ingredients in the dietary supplement formulation, which have strong antioxidant and anti-inflammatory effects, promote the repair of acinar epithelial and peripheral nerve damage. Ares 2 formula contains vitamin C, vitamin E, beta carotene, zinc, and copper, which are similar to the dietary supplement we used in this study. Eskina et al. observed the improvement of visual quality by using the drug complex responding to the Ares 2 formula (29). Told et al. confirmed that dietary supplement Retaron^®^ can play an antioxidant role in the human body, thus affecting the retinal dynamic function (30). However, their study did not assess the therapeutic effect of the dietary supplement on the ocular surface microenvironment.

Among the components in the dietary supplement we tested in this study, the high concentration of polyphenol compounds and cyanidin-3-O-glucoside chloride in aronia extracts can quickly and effectively remove oxygen free radicals and play a strong antioxidant effect (31). In addition, aronia extract contains active components such as nitric oxide synthase-2 and cyclooxygenase-2, which can effectively inhibit inflammatory and oxidative reactions (32). Cavet et al. also confirmed that polyphenols have anti-inflammatory and antioxidant effects on human corneal epithelial cells, which can be used to treat eye inflammation caused by diseases and have a potential value in the treatment of dry eyes (33).

Omega-3 free fatty acids can optimize the transformation of meibum from saturated fatty acids to unsaturated fatty acids under pathological conditions (4), so it may effectively promote the generation and excretion of meibomian esters, improve the obstruction of glandular ducts, and reduce the local inflammatory reaction, thus restoring the structure of MG. By improving the properties of eyelid esters and reducing the inflammation at the edge of the eyelid, the stability of the lipid layer of the tear film should be gradually restored, and therefore, the symptoms and signs of dry eyes could be indirectly improved and eventually improve the visual quality. Macsai evaluated the ocular symptoms of MGD patients and found that the use of omega-3 fatty acids improved the scores of OSDI, TBUT, and meibomian, which was similar to the results of our study (34). Meanwhile, Deinema et al. also confirmed that omega-3 fatty acids can reduce tear osmolality and improve tear film stability in patients with dry eye, thus improving the symptoms of dry eye (35). However, our results showed that there were no obvious advantages in the improvement of meibomian ester and MG excretion after dietary supplement was added, which might be related to the short-term treatment and observation period, and small sample size in this study. We will increase the sample size and extend the study period in the future, which may lead to more representative and conclusive results.

In summary, for the treatment of MGD-related dry eye that is based on conventional treatment strategies, the addition of dietary supplement can further improve the symptoms and signs of dry eye, reduce the inflammatory response of MG, restore the glandular structure, and improve the visual quality, without any systemic or local adverse reactions. However, there were no significant advantages in the improvement of meibomian ester and MG excretion. Dietary supplement is expected to be a safe and effective systemic drug in the treatment of MGD-related dry eye.

## Data Availability Statement

The original contributions presented in the study are included in the article/supplementary material, further inquiries can be directed to the corresponding author/s.

## Ethics Statement

This study followed the Declaration of Helsinki, conformed to the principles of medical ethics, and was approved by the Ethics Committee of Xiangya Hospital of Central South University (Document Number: 201912528). The patients/participants provided their written informed consent to participate in this study.

## Author Contributions

KL and YC: data acquisition and/or research execution, data analysis and/or interpretation, and manuscript preparation. XP and ML: data acquisition and/or research execution, data analysis and/or interpretation. RY: data analysis and/or interpretation, manuscript preparation. JL, XT, and YX: data acquisition and/or research execution. YC and QL: data analysis and/or interpretation. HW: research design, data acquisition and/or research execution, data analysis and/or interpretation, and manuscript preparation. All authors contributed to the article and approved the submitted version.

## Funding

This study was supported by National Natural Science Foundation of China (No. 81170823), Natural Science Found of Hunan Province (No. 2019JJ40502), Science and Technology Project of Hunan Administration of Traditional Chinese Medicine (No. 201856), Central Committee Guides Local Science and Technology Development Special Project by Ministry of Science and Technology (No. 2020YDDF0043), and Major Special Project of Key Research and Development Program of Ningxia Province (No. 2021BEG01001).

## Conflict of Interest

The authors declare that the research was conducted in the absence of any commercial or financial relationships that could be construed as a potential conflict of interest.

## Publisher's Note

All claims expressed in this article are solely those of the authors and do not necessarily represent those of their affiliated organizations, or those of the publisher, the editors and the reviewers. Any product that may be evaluated in this article, or claim that may be made by its manufacturer, is not guaranteed or endorsed by the publisher.
